# Extent and Incidence of Pseudo‐Worsening of Kidney Function Due to Oral Antitumor Therapeutics in the AMBORA Cohort: An Analysis of Real‐World Data

**DOI:** 10.1002/cpt.70166

**Published:** 2025-12-22

**Authors:** Michael I. Sponfeldner, Pauline Dürr, Phyllis Lensker, Katja Gessner, Lisa Cuba, Rainer Fietkau, Markus F. Neurath, Bernd Wullich, Marianne Pavel, Carola Berking, Matthias W. Beckmann, Andreas Mackensen, Frank Dörje, Martin F. Fromm

**Affiliations:** ^1^ Institute of Experimental and Clinical Pharmacology and Toxicology Friedrich‐Alexander‐Universität Erlangen‐Nürnberg Erlangen Germany; ^2^ Pharmacy Department Erlangen University Hospital and Friedrich‐Alexander‐Universität Erlangen‐Nürnberg Erlangen Germany; ^3^ Comprehensive Cancer Center Erlangen‐EMN Uniklinikum Erlangen Erlangen Germany; ^4^ Bavarian Cancer Research Center (BZKF) Erlangen Germany; ^5^ Department of Radiation Oncology Uniklinikum Erlangen and Friedrich‐Alexander‐Universität Erlangen‐Nürnberg Erlangen Germany; ^6^ Department of Medicine 1, Gastroenterology, Pneumology and Endocrinology Uniklinikum Erlangen and Friedrich‐Alexander‐Universität Erlangen‐Nürnberg Erlangen Germany; ^7^ Department of Urology and Paediatric Urology Uniklinikum Erlangen and Friedrich‐Alexander‐Universität Erlangen‐Nürnberg Erlangen Germany; ^8^ Department of Dermatology Uniklinikum Erlangen and Friedrich‐Alexander‐Universität Erlangen‐Nürnberg Erlangen Germany; ^9^ Department of Obstetrics and Gynaecology Uniklinikum Erlangen and Friedrich‐Alexander‐Universität Erlangen‐Nürnberg Erlangen Germany; ^10^ Department of Internal Medicine 5, Hematology and Oncology Uniklinikum Erlangen and Friedrich‐Alexander‐Universität Erlangen‐Nürnberg Erlangen Germany; ^11^ FAU NeW – Research Center New Bioactive Compounds Friedrich‐Alexander‐Universität Erlangen‐Nürnberg Erlangen Germany; ^12^ Present address: Pharmacy Department Clinic Floridsdorf, Vienna Healthcare Group Vienna Austria

## Abstract

A considerable number of oral antitumor therapeutics (OAT) has the potential for causing pseudo‐worsening of kidney function (PW) due to inhibition of renal creatinine secretion, i.e., kidney function is unaffected, while creatinine‐based calculation of glomerular filtration rate (eGFR) erroneously indicates an impaired kidney function. Nonrecognition of PW can lead to dose reductions, interruptions, or discontinuations of OAT. The extent and incidence of PW has so far not been studied for a broad set of OAT in clinical routine. In this retrospective, multicenter cohort study, 694 AMBORA patients newly started on OAT were assessed for eligibility. eGFR values were compared between baseline and within 30 days of treatment. OAT were separated into the groups unlikely causing and likely causing/with proven PW. The usage of cystatin C measurements as an alternative method for assessing kidney function was evaluated. A total of 238 patients received 38 OAT likely causing PW/with proven PW. In this group, eGFR decreased significantly (−6.8 ml/min, *p* < 0.001). In 17.2% of these patients, eGFR decreased by ≥20 ml/min. Significant decreases in eGFR were observed for patients receiving, for example, abemaciclib, ribociclib, and osimertinib. Cystatin C measurements were not performed in 95.8% of the patients. In the group of 67 patients receiving OAT unlikely causing PW, there was no significant change in eGFR. In clinical routine, multiple OAT associated with PW are prescribed. A very low rate of usage of creatinine‐independent methods for assessing kidney function (cystatin C) indicates that further training of oncologists is required on OAT‐induced PW to further improve patient safety.

**Study Highlights**

**WHAT IS THE CURRENT KNOWLEDGE ON THE TOPIC?**

Multiple oral antitumor therapeutics cause pseudo‐worsening of kidney function, that is, kidney function is unaffected, while creatinine‐based calculation of glomerular filtration rate (eGFR) erroneously indicates an impaired kidney function.

**WHAT QUESTION DID THIS STUDY ADDRESS?**

What is the extent and incidence of pseudo‐worsening of kidney function for a broad set of oral antitumor therapeutics in clinical routine care?

**WHAT DOES THIS STUDY ADD TO OUR KNOWLEDGE?**

This study shows that a considerable number of oral antitumor therapeutics associated with pseudo‐worsening of kidney function is prescribed in clinical routine, causing eGFR decreases to a variable extent. Creatinine‐independent methods of kidney function assessment, such as eGFR based on cystatin C, are only very rarely used.

**HOW MIGHT THIS CHANGE CLINICAL PHARMACOLOGY OR TRANSLATIONAL SCIENCE?**

Bringing together the fields of transporter‐mediated renal creatinine secretion and medication safety in oncology provides the basis for improved care of patients treated with oral antitumor therapeutics.

Following the introduction of the tyrosine kinase inhibitor (TKI) imatinib in 2001, more than 100 different oral antitumor therapeutics (OAT) have been approved.^1^, ^2^ The use of OAT is rapidly increasing and is associated with considerable benefits for patients, for example, improved outcomes and convenience of application in comparison to intravenously administered drugs.^3^

Among other factors, close consideration of kidney function is crucial for treatment response and success in cancer therapy.^4^, ^5^ Renal insufficiency is frequently observed in cancer patients due to their age, various comorbidities, or nephrotoxicity of certain antitumor drugs.^4^, ^6^ Thus, regular and close monitoring of kidney function in cancer patients is clinical practice.^4^, ^6^

Kidney function is commonly assessed by measuring serum creatinine concentrations (SCr) and subsequent calculation of estimated glomerular filtration rate (eGFR_SCr_) using established equations.^7^, ^8^, ^9^ In addition to passive glomerular filtration, creatinine is actively secreted into urine by several renal transport proteins (OCT2, organic cation transporter 2; MATE1, MATE2‐K; multidrug and toxin extrusion proteins 1 and 2‐K).^10^, ^11^, ^12^, ^13^ Elevated SCr usually reflects a reduction of eGFR_SCr_ and is used as an indicator for renal insufficiency or kidney failure.^14^, ^15^ In cases of serum creatinine being considered an unreliable marker for kidney function assessment, cystatin C (CysC), which is filtered by the kidneys, is commonly used as an alternative in clinical practice.^15^, ^16^ It has been previously shown that a considerable number of OAT, primarily TKI, inhibit renal creatinine transporters, resulting in increased SCr and decreased eGFR_SCr_ without inducing kidney damage, as shown by alternative methods of kidney function assessment such as cystatin C‐based eGFR (eGFR_CysC_). These drugs cause pseudo‐worsening of kidney function.^17^, ^18^, ^19^, ^20^, ^21^, ^22^ If unrecognized by treating physicians, this poses patients at considerable risks for severe medication errors such as unnecessary OAT dose reductions, treatment interruptions, or discontinuations.^23^ Moreover, we recently showed that prescribing information or Summaries of Product Characteristics (SmPCs) of multiple OAT are of insufficient quality to provide physicians with precise and clinically useful information on the issue of pseudo‐worsening of kidney function.^17^

There is still limited knowledge on the extent and incidence of pseudo‐worsening of kidney function due to renal creatinine transporter inhibition during routine clinical conditions.^19^, ^20^, ^24^, ^25^, ^26^, ^27^ Most of the previous studies focused on selected, individual OAT administered to healthy volunteers or in patients with specific cancer entities such as lung cancer.^23^, ^25^ Despite the high clinical relevance of this effect, a broad evaluation of pseudo‐worsening of kidney function in real‐world conditions during treatment with OAT is still missing.

The aim of our study was therefore a retrospective evaluation of clinical real‐world data on the incidence and extent of pseudo‐worsening of kidney function caused by a broad range of different OAT in patients suffering from various cancer entities, enrolled in the randomized, multicenter AMBORA trial^28^ and the AMBORA Center.^2^, ^29^

## MATERIALS AND METHODS

### Data collection

Data were retrospectively collected from patients enrolled in the randomized, multicenter AMBORA trial^28^ and the AMBORA Center.^29^ This group is termed the AMBORA cohort in the following. In brief, the AMBORA trial successfully investigated the outcomes of an intensified clinical pharmacological/pharmaceutical care program over 12 weeks after the initiation of OAT for patients with a broad range of cancer entities.^28^ This was followed by the implementation of this standardized care program into clinical routine within the AMBORA Center at the Comprehensive Cancer Center (CCC) Erlangen‐EMN.^2^, ^29^ Patient data collection in the AMBORA cohort adhered to standard operating procedures and included structured longitudinal collection of demographic and clinical data.^2^, ^28^, ^29^

For this analysis, we extracted demographic and clinical data (i.e., cancer entity, oncological therapy regimen, co‐medications at OAT initiation, OAT interruptions, and discontinuations) from the AMBORA cohort for the time period between OAT initiation and week 12 of treatment. Additional laboratory values were extracted from the AMBORA database or the electronic health records (EHR) for up to 12 weeks after OAT initiation. Furthermore, EHR were screened for information on previous kidney transplantation (NTx), nephrotoxicity, or other descriptions of kidney injury during the 12‐week period.

Approvals were obtained by the ethics committee of the Friedrich‐Alexander Universität Erlangen‐Nürnberg. The AMBORA projects were registered at the German Clinical Trials Register (DRKS00013271/DRKS00026272). The present study was conducted in accordance with the STROBE and ESMO‐GROW guidelines as far as items were applicable and all patients provided informed consent.

### Data analysis

#### Eligibility criteria

**Figure 1:**
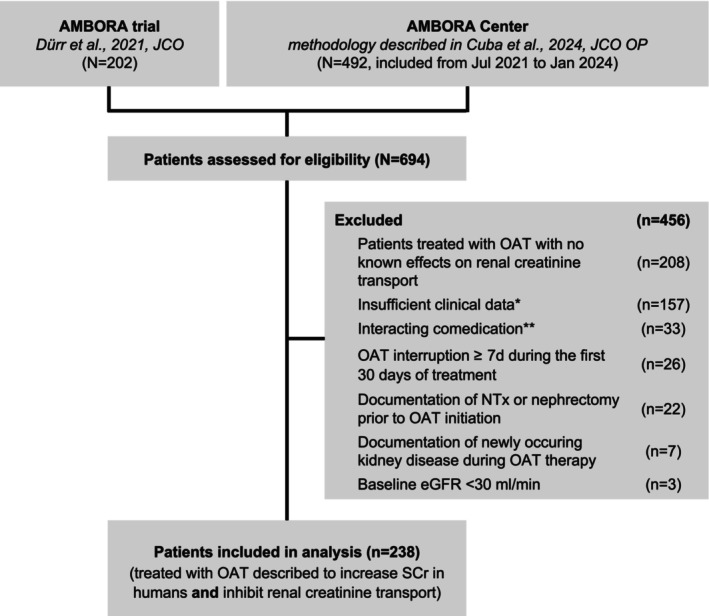
Flow diagram for included patients treated with oral antitumor therapeutics likely causing or proven to cause pseudo‐worsening of kidney function. * = insufficient clinical data corresponding to no serum creatinine concentration available at baseline, no follow‐up serum creatinine concentration available, <2 serum creatinine concentrations available within 7 days of initiation of the oral antitumor therapeutic, no baseline co‐medications available; ** = co‐medications also described to cause pseudo‐worsening of kidney function (e.g., trimethoprim) or being nephrotoxic. eGFR, estimated glomerular filtration rate; NTx = kidney transplantation; OAT, oral antitumor therapeutic; SCr, serum creatinine concentration.

Patients from the AMBORA cohort were included if sufficient clinical data were available [i.e., a baseline SCr/eGFR_SCr_ measurement prior to OAT initiation (eGFR_SCr_ ≥ 30 ml/min) and ≥1 subsequent measurement of SCr/eGFR_SCr_ within 30 days of OAT initiation or ≥1 subsequent measurement of SCr prior to drop out, if drop out occurred within 30 days of OAT initiation, information on co‐medications at OAT initiation]. Patients were excluded if they previously underwent NTx/nephrectomy or had documentation of newly occurring kidney injury/nephrotoxicity in EHR within 12 weeks of OAT initiation. If patients interrupted their OAT therapy ≥7 days within 30 days of OAT initiation, they were also excluded. In addition, patients were excluded if they newly initiated co‐medications potentially confounding the effect of the OAT on serum creatinine concentrations, that is, a non‐antitumor drug that causes pseudo‐worsening of kidney function (e.g., trimethoprim) or other drugs known to alter serum creatinine (e.g., ACE inhibitors). An eGFR_SCr_ ≥ 30 ml/min was chosen as cutoff for eligibility to minimize outcome misclassification, since patients with an eGFR_SCr_ < 30 ml/min are more prone to true acute kidney injury due to other reasons. A respective flow chart is shown in **Figure** **1**.

Analyses were performed for the following groups of patients receiving OAT: (1) likely causing pseudo‐worsening of kidney function, (2) with proven pseudo‐worsening of kidney function,^17^, ^21^ and (3) as a comparison group (**Figure** **S1**), receiving OAT not described in the literature to inhibit renal creatinine transporters, i.e., which are unlikely to cause pseudo‐worsening of kidney function.

#### Calculation of eGFR

For patients enrolled in the AMBORA trial, eGFR_SCr_ was primarily calculated using the Modification of Diet in Renal Disease (MDRD) formula. Due to changes in clinical chemistry reporting for patients enrolled in the AMBORA Center, the Chronic Kidney Disease Epidemiology Collaboration (CKD‐EPI) formula was used.^7^ In order to have comparable eGFR_SCr_ for all patients, eGFR_SCr_ was calculated using the CKD‐EPI formula for all patients with MDRD‐based eGFR_SCr_ available. For eGFR_CysC_, the corresponding CKD‐EPI formula using CysC instead of SCr was used for all patients with available CysC measurements.^7^

#### Assessment of the potential for nephrotoxicity

Since nephrotoxicity induced by OAT may serve as an alternative explanation for alterations in SCr, the corresponding German SmPCs of the OAT likely causing or proven to cause pseudo‐worsening of kidney function (most recent, preferably original manufacturer, accessed via fachinfo.de)^30^ included in our study were systematically evaluated for information regarding the description of nephrotoxicity as a side effect.

#### Extent and incidence of changes in eGFR_SCr_ and incidence of CysC measurements

The maximum absolute changes in SCr and eGFR_SCr_ within 30 days of OAT initiation were evaluated for all included patients, including stratified analyses based on baseline eGFR_SCr_ (≥ 90 ml/min, 89–60 ml/min, 59–30 ml/min). Additionally, a stratified analysis for the individual OAT (≥3 patients per OAT in the dataset) was performed. Moreover, decreases in eGFR_SCr_ were analyzed regarding their extent, that is, <10 ml/min, 10–19 ml/min, 20–29 ml/min, ≥30 ml/min, for all OAT. eGFR_SCr_ decreases ≥10 ml/min were considered clinically relevant.

In addition, an analysis of the long‐term changes in kidney function beyond the 30‐day observation period was performed. For this purpose, the maximum changes in eGFR_SCr_ among patients receiving continuous OAT and the maximum declines among those patients receiving OAT with cyclic dosing schemes from day 31 to within 12 weeks were evaluated. These changes in eGFR_SCr_ were then compared with the maximum change in eGFR_SCr_ within 30 days of OAT initiation.

Furthermore, the incidence of concomitant CysC measurements, that is, at OAT initiation and/or subsequent timepoints, within the 30‐day time period was analyzed.

#### Statistical analyses

Baseline characteristics, extent and incidence of decreases in eGFR_SCr_, as well as comparison of eGFR_SCr_/eGFR_CysC_ were analyzed using descriptive statistics. For evaluation of changes in SCr and eGFR_SCr_, the maximum change per patient within 30 days and within 12 weeks of OAT initiation was identified and pooled for overall comparisons. Additionally, stratified analyses for individual OAT were performed. Paired *t*‐tests were used for testing for statistical significance (*p* < 0.05) in normally distributed variables and Wilcoxon signed‐rank test for nonnormally distributed variables for each OAT. For comparison of the changes in eGFR_SCr_ between day 31 and week 12 with the maximum change in eGFR_SCr_ within 30 days, a repeated measures ANOVA including post hoc paired comparisons was performed. Since our analyses were explorative, no adjustment for multiple testing was performed. Statistical analyses were performed using R (Version 4.4.1, R Foundation for Statistical Computing, Vienna, Austria) and Microsoft Excel™ (Version 2021, Microsoft Corporation, Redmond, USA).

## RESULTS

### Patients

In total, 238 patients treated with 38 different OAT likely causing or with proven pseudo‐worsening of kidney function were included (**Figure** **1**). Of these, 114 patients (47.9%) received OAT (*n* = 18/38, 47.4%) proven to cause pseudo‐worsening of kidney function.^17^, ^21^ Baseline demographic and clinical characteristics of the 238 patients are provided in **Table** **1**. A detailed overview of individual OAT prescribed to the 238 patients is shown in **Table** **S1**. As a comparison group, 67 patients treated with OAT unlikely to cause pseudo‐worsening of kidney function were included (**Figure** **S1**, **Table** **S2**).

**Table 1:** Baseline characteristics of included 238 patients treated with OAT likely causing or proven to cause pseudo‐worsening of kidney function

Characteristic	No. [%]
Age [years] (mean, range)	63.9 (28–92)
Sex [female]	141 [59.2%]
**Cancer type** a	
Solid tumors	
Breast cancer	57 [23.9%]
Melanoma	35 [14.7%]
Renal cell carcinoma	29 [12.2%]
Neuroendocrine tumors (pancreas, lung, small intestine)	15 [6.3%]
Bronchial carcinoma	13 [5.5%]
Prostate cancer	11 [4.6%]
Hepatocellular carcinoma	7 [2.9%]
Thyroid cancer	7 [2.9%]
Soft tissue sarcoma	6 [2.5%]
Ovarian cancer	5 [2.1%]
Systemic mastocytosis	5 [2.1%]
Hematologic malignancies	
Chronic myeloid leukemia	12 [5.0%]
Acute myeloid leukemia	9 [3.8%]
Other	27 [11.3%]
**Oral antitumor therapeutic** b	
Cabozantinib	32 [13.5%]
Dabrafenib + trametinib	28 [11.8%]
Ribociclib	23 [9.6%]
Abemaciclib	15 [6.3%]
Olaparib	15 [6.3%]
Palbociclib	14 [5.9%]
Midostaurin	12 [5.0%]
Pazopanib	10 [4.2%]
Encorafenib + binimetinib	9 [3.8%]
Nilotinib	9 [3.8%]
Lenvatinib	8 [3.4%]
Darolutamide	7 [2.9%]
Osimertinib	6 [2.5%]
Sunitinib	6 [2.5%]
Imatinib	5 [2.1%]
Axitinib	4 [1.7%]
Crizotinib	3 [1.3%]
Niraparib	3 [1.3%]
Regorafenib	3 [1.3%]
Tivozanib	3 [1.3%]
Other (18 drugs)	23 [9.6%]
Dosage scheme	
Cyclic	39 [16.4%]
Continuous	199 [83.6%]
Creatinine	
Baseline [mg/ml] (mean, range)	0.89 (0.39–1.69)
eGFR_SCr_	
Baseline [ml/min] (mean, range)	82.2 (33–129)
Baseline ≥90 ml/min	102 [42.9%]
Baseline 60–89 ml/min	93 [39.0%]
Baseline 30–59 ml/min	43 [18.1%]

^a^ Only cancer types with ≥5 patients in the dataset are listed in this table; the remaining entities were grouped within the category ‘other’.

^b^ Only drugs taken by ≥3 patients in the dataset are listed in this table; the remaining entities were grouped within the category ‘other’.

### Overall changes in eGFR_SCr_ within 30 days of OAT initiation

**Figure 2:**
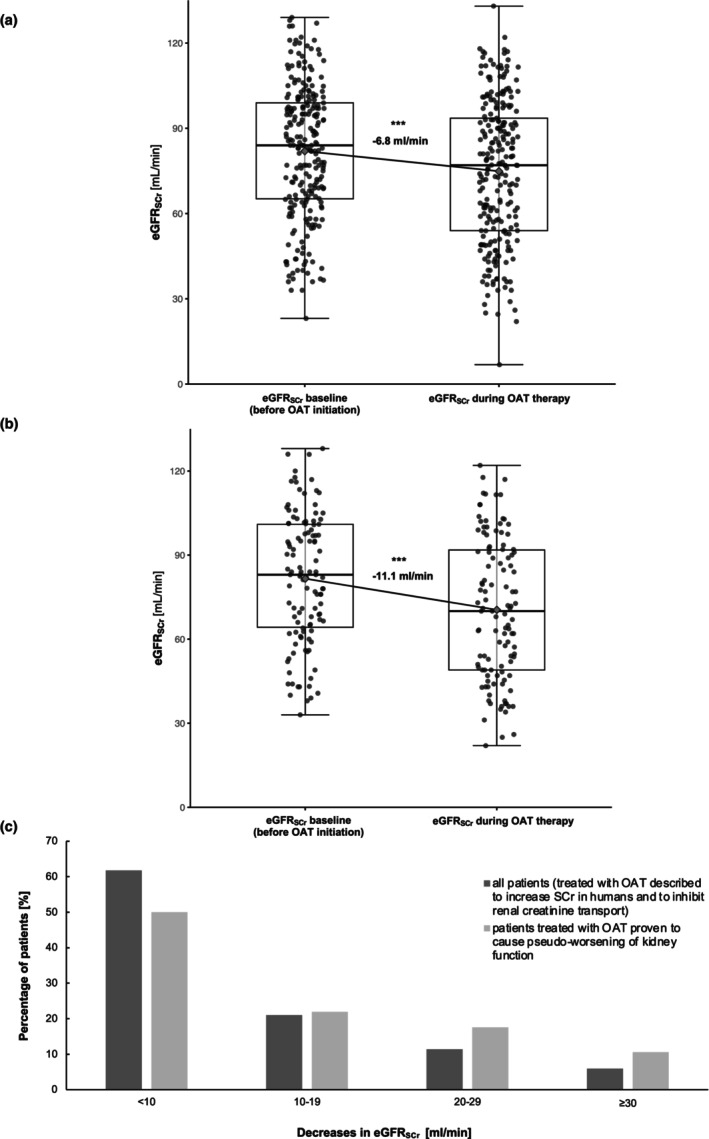
Serum creatinine‐based eGFR in patients newly treated with oral antitumor therapeutics before oral antitumor therapeutic initiation and within 30 days. (a) Analysis of 238 patients treated with oral antitumor therapeutics likely causing or proven to cause pseudo‐worsening of kidney function, (b) Subgroup analysis of 114 patients treated with oral antitumor therapeutics proven to cause pseudo‐worsening of kidney function, (c) Frequency distribution of the extent of eGFR decreases for the 238 patients treated with oral antitumor therapeutics likely causing or proven to cause pseudo‐worsening of kidney function (dark gray) and the subgroup of 114 patients treated with OAT proven to cause pseudo‐worsening of kidney function (light gray). Box plot: dots, data for individual patients with random jittering for better graphical representation; gray square, mean. eGFR, estimated glomerular filtration rate; OAT, oral antitumor therapeutic; SCr, serum creatinine concentration. ***p < 0.001.

In the 238 patients treated with OAT likely causing or proven to cause pseudo‐worsening of kidney function, eGFR_SCr_ decreased by −6.8 ml/min ± 14.5 ml/min [95% CI, −5.0 to −8.6 ml/min, *p* < 0.001] (**Figure** **2** **a**). For the subgroup of 114 patients treated with OAT proven to cause pseudo‐worsening of kidney function, eGFR_SCr_ decreased by −11.1 ml/min ± 14.7 ml/min [95% CI, −8.4 to −13.8 ml/min, *p* < 0.001] (**Figure** **2** **b**). The comparison group of 67 patients treated with OAT unlikely to cause pseudo‐worsening of kidney function had no significant mean change in eGFR_SCr_ (−1.0 ml/min ± 7.28 ml/min; 95% CI: −2.7 to −13.8 ml/min, n.s.) (**Figure** **S2**). In the stratified analysis for different baseline eGFR_SCr_ levels, eGFR_SCr_ decreased by −9.9 ml/min (baseline eGFR_SCr_ ≥ 90 ml/min, *p* < 0.001), by −5.8 ml/min (baseline eGFR_SCr_ 89–60 ml/min,  *p*< 0.001), and by −1.6 ml/min (baseline eGFR_SCr_ 59–30 ml/min, n.s.), respectively (**Figure** **S3A–C**).

The distribution of the extent of decreases in eGFR_SCr_ is shown in **Figure** **2** **c**. For the 238 patient cohort treated with OAT likely causing or proven to cause pseudo‐worsening of kidney function, eGFR_SCr_ decreased ≥20 ml/min in 17.2% of patients. For the 114 patient subgroup treated with OAT proven to cause pseudo‐worsening of kidney function, eGFR_SCr_ decreased ≥20 ml/min in 28.0% of patients. The largest observed decrease in eGFR_SCr_ was −52 ml/min in a patient treated with ribociclib. Concerning clinically relevant eGFR_SCr_‐thresholds of 60 ml/min and 30 ml/min, in the 238 patient cohort eGFR_SCr_ decreased below 60 ml/min in 34 patients (14.3%) and below 30 ml/min in 5 patients (2.1%). In the 114 patient subgroup, eGFR_SCr_ decreased below 60 ml/min in 25 patients (21.9%) and below 30 ml/min in three patients (2.6%).

### Extent of changes in eGFR_SCr_ at the level of individual OAT

**Figure 3:**
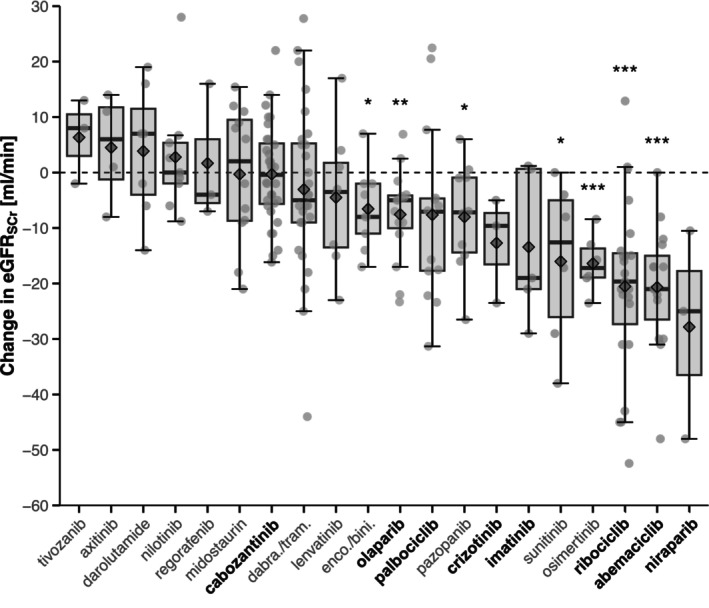
Serum creatinine‐based eGFR changes in patients newly treated with oral antitumor therapeutics likely causing or proven to cause pseudo‐worsening of kidney function stratified for individual oral antitumor therapeutics (≥3 patients/OAT in the population of 238 patients). OAT in bold are proven to cause pseudo‐worsening of kidney function. Box plot: dots, data for individual patients with random jittering for better graphical representation; gray square, mean. eGFR, estimated glomerular filtration rate; dabra/tram, dabrafenib + trametinib, enco/bini = encorafenib + binimetinib; OAT, oral antitumor therapeutic; SCr, serum creatinine concentration. *p < 0.05, **p < 0.01, ***p < 0.001.

The extent of changes in eGFR_SCr_ for the individual OAT with ≥3 patients in the 238 patient cohort receiving OAT likely causing or proven to cause pseudo‐worsening of kidney function within 30 days of OAT initiation is shown in **Figure** **3**. Patients treated with niraparib had the largest mean decreases in eGFR_SCr_ (−27.8 ml/min ± 18.9 ml/min, 95% CI: −49.2 to −6.4 ml/min, *p* = 0.126, *n* = 3), followed by abemaciclib (−20.6 ml/min ± 11.3 ml/min, 95% CI: −26.4 to −14.9 ml/min, *p* < 0.001, *n* = 15), and ribociclib (−20.5 ml/min ± 15.8 ml/min, 95% CI: −26.9 to −14.1 ml/min, *p* < 0.001, *n* = 23). The respective extent of corresponding changes in SCr for the individual OAT is provided in **Figure** **S4**. For OAT with <3 patients in the dataset, the changes in eGFR_SCr_ are shown in **Figure** **S5**.

### Incidence of decreases in eGFR_SCr_ at the level of individual OAT

**Figure 4:**
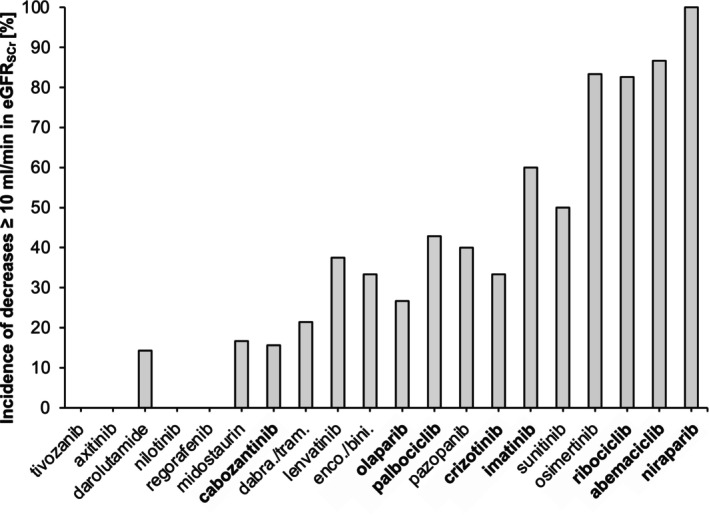
Incidence of serum creatinine‐based eGFR decreases ≥10 ml/min in patients newly treated with oral antitumor therapeutics likely causing or proven to cause pseudo‐worsening of kidney function stratified for individual oral antitumor therapeutics (≥3 patients/OAT in the population of 238 patients). OAT in bold are proven to cause pseudo‐worsening of kidney function. The order of the oral antitumor therapeutics corresponds to the order in Figure 3. eGFR, estimated glomerular filtration rate; dabra/tram, dabrafenib + trametinib; enco/bini, encorafenib + binimetinib; OAT, oral antitumor therapeutic; SCr, serum creatinine concentration.

The incidence of decreases ≥10 ml/min in eGFR_SCr_ for the individual OAT with ≥3 patients in the 238 patient cohort within 30 days of OAT initiation is shown in **Figure** **4**. In agreement with the extent of changes in eGFR_SCr_, the highest incidence of decreases was observed for niraparib (100%, *n* = 3/3), followed by abemaciclib (86.7%, *n* = 13/15) and osimertinib (83.3%, *n* = 5/6).

### Comparison of changes in eGFR_SCr_ within 30 days and from day 31 to week 12 of OAT initiation

In the 238 patients treated with OAT likely causing or proven to cause pseudo‐worsening of kidney function, eGFR_SCr_ decreased by 5.9 ml/min ± 16.4 ml/min [95% CI: −3.9 to −8.0 ml/min, *P* < 0.001] from day 31 to week 12 of OAT initiation in comparison to baseline. No significant difference was observed (+0.9 ml/min, n.s.) when comparing the change from baseline to within 30 days and from day 31 to week 12 (**Figure** **S6**).

### Incidence of cystatin C measurements

**Figure 5:**
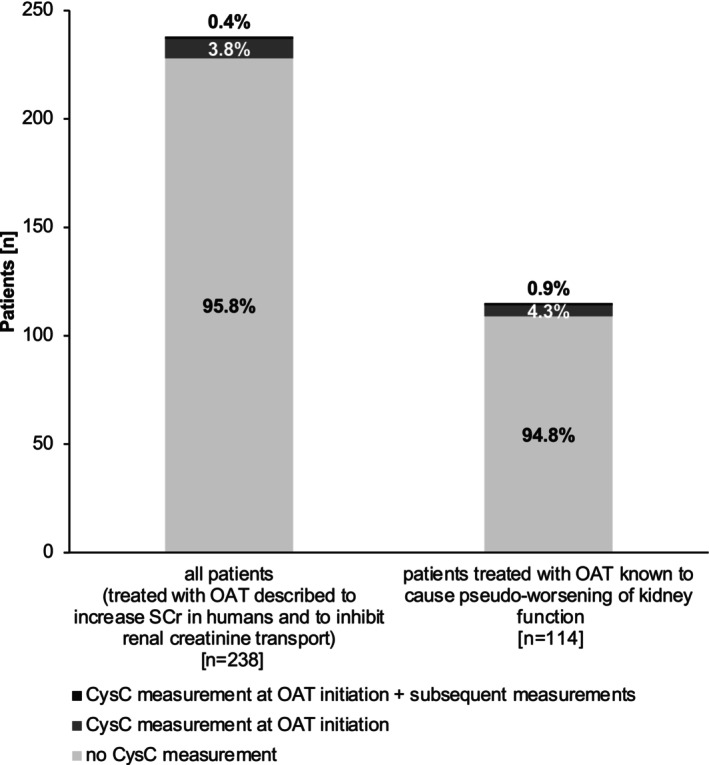
Incidence of cystatin C measurements in 238 patients treated with oral antitumor therapeutics likely causing or proven to cause pseudo‐worsening of kidney function and in the subgroup of 114 patients treated with oral antitumor therapeutics proven to cause pseudo‐worsening of kidney function before and within 30 days of oral antitumor therapeutic initiation. The light gray of the bars corresponds to no cystatin C measurement within 30 days of OAT initiation. The dark gray of the bars corresponds to a cystatin C measurement before/at OAT initiation, but no subsequent cystatin C measurement is available. The black area of the bars corresponds to a cystatin C measurement at OAT initiation and ≥1 subsequent cystatin C measurement within 30 days of OAT initiation. eGFR, estimated glomerular filtration rate; OAT, oral antitumor therapeutic; SCr, serum creatinine concentration.

The incidence of CysC measurements at OAT initiation with and without subsequent measurements within 30 days is shown in **Figure** **5**. There was no notable difference between the 238 patient cohort treated with OAT likely causing or proven to cause pseudo‐worsening of kidney function and the 114 patient cohort treated with OAT known to cause pseudo‐worsening of kidney function, with no CysC measurements during the observation period in 95.8% and 94.8% of patients, respectively. A comparison of eGFR_SCr_ and eGFR_CysC_ measurements is given in **Table** **S3**.

### Assessment of the potential for nephrotoxicity

In total, SmPCs of 16/38 (42%) OAT likely causing or proven to cause pseudo‐worsening of kidney function, used in our patient cohort, had warnings regarding kidney function, of which 1/38 (2.6%, vemurafenib) explicitly stated the risk of nephrotoxicity (i.e., risk of ‘acute tubular necrosis’ and ‘acute interstitial nephritis’) and 8/38 (21%) stated the potential for kidney failure without stating frequencies. SmPCs of 21/38 (55.3%) OAT listed renal adverse drug reactions, for example, acute kidney injury, kidney insufficiency or kidney failure. These different renal adverse drug reactions were described as ‘frequent’ (≥1% to <10%) in 8 SmPCs, ‘sometimes’ (≥0.1% to <1%) in 17 SmPCs and ‘rarely’ (≥0.01% to <0.1%) or ‘of unknown frequency’ in 6 SmPCs.

## DISCUSSION

This is the first study evaluating pseudo‐worsening of kidney function across a broad range of different OAT in patients with various cancer entities in a clinical real‐world setting without any restrictions regarding OAT class or tumor entity. The major findings are that (1) a considerable number of OAT are associated with decreases in eGFR_SCr_, (2) the extent of decreases in eGFR_SCr_ is pronounced in a substantial number of cases, i.e., 17.2% of patients treated with OAT likely causing or proven to cause pseudo‐worsening of kidney function (*n* = 41/238) and 28.0% of patients treated with OAT proven to cause pseudo‐worsening of kidney function (*n* = 32/114) experienced decreases in eGFR_SCr_ ≥ 20 ml/min and (3) alternative creatinine‐independent methods of kidney function assessment such as eGFR_CysC_ are used in less than 5% of the patients indicating a possible lack of knowledge of physicians on this effect.

It was already shown that patients treated with various OAT known to inhibit renal creatinine secretion show discrepancies between creatinine‐based kidney function assessments and other, “gold‐standard” assessments (e.g., using CysC or iohexol), i.e., these patients are affected by pseudo‐worsening of kidney function.^23^, ^24^, ^25^, ^26^, ^27^, ^31^, ^32^, ^33^ However, the transferability of these findings to clinical practice is limited, since studies were performed in healthy volunteers, findings were based solely on case reports or focused on specific cancer entities.^23^, ^25^ Moreover, as we showed in a previous study, the corresponding prescribing information and SmPCs of multiple OAT proven to cause pseudo‐worsening of kidney function provide insufficient quality of information to adequately inform clinical oncological decision making, posing patients at risk for severe medication errors.^17^

In agreement with previous studies focusing on selected OAT used for the treatment of gynecological malignancies,^23^, ^31^ the results of this study show the largest mean decreases in eGFR_SCr_ as well as the highest incidences of decreases in eGFR_SCr_ for niraparib, abemaciclib, and ribociclib, with also considerable mean eGFR_SCr_ decreases as well as a high incidence of decreases observed for olaparib and palbociclib. Thus, pseudo‐worsening of kidney function is of considerable relevance, especially in gynecologic oncology.

Next to OAT already known to cause pseudo‐worsening of kidney function, the results of this study also show significant mean decreases in eGFR_SCr_ for OAT such as osimertinib, sunitinib, or pazopanib, which have been shown to interact with renal creatinine transporters.^17^ However, the lack of CysC measurements in the literature, SmPCs, and our retrospective dataset precludes definitive confirmation of pseudo‐worsening of kidney function for these OAT. Nevertheless, the results of this study provide a basis for future investigations to validate pseudo‐worsening of kidney function for these OAT.

When comparing the changes in eGFR_SCr_ within 30 days of OAT initiation and from day 31 to week 12, no significant difference was observed, indicating constant and persistent pseudo‐worsening of kidney function and no further decline in kidney function after the initial decline at OAT initiation. These findings support the assumption that patients treated with OAT experience pseudo‐worsening of kidney function rather than true acute kidney injury or nephrotoxicity.

The results of this study also demonstrate considerable variability of decreases in eGFR_SCr_ within patients treated with the same OAT and the same dose. For example, observed decreases in eGFR_SCr_ ranged from 0 ml/min to −48 ml/min for the CDK4/6 inhibitor abemaciclib. Possible explanations for this observed variability are (1) interindividual differences in creatinine transporter expression and function, (2) interindividual differences in plasma concentrations of OAT (e.g., due to variable absorption, metabolism),^34^, ^35^ and (3) interindividual differences in adherence to the OAT.

Despite previously addressed, already available evidence on pseudo‐worsening of kidney function, creatinine‐independent methods of kidney function assessment such as eGFR_CysC_ were used in less than 5% of patients in our cohort. Notably, there were no differences between the overall study population and patients treated with OAT known to cause this effect. Since eGFR_CysC_ provides a relatively inexpensive, viable alternative for eGFR_SCr_ in clinical routine care, it appears that oncologists need to be made more aware of the issue of OAT‐induced pseudo‐worsening of kidney function to avoid medication errors such as unnecessary OAT dose reductions, OAT interruption, OAT discontinuation, or modifications of co‐medications.

## LIMITATIONS

The following limitations of this study have to be considered: Since this was a retrospective cohort study, the evaluations were dependent on the data available. As in other cohort studies utilizing previously collected data, this introduces the issue of potentially insufficient data quality. However, as the data used were derived from standardized clinical documentation, that is, data from the AMBORA cohort^2^, ^28^, ^29^ as well as electronic healthcare data, we consider the risk of bias thereof to be low.

Furthermore, the dataset included only a very limited number of CysC measurements, thereby precluding differentiation of eGFR_SCr_ decreases into pseudo‐worsening of kidney function or real kidney injury. However, we also investigated corresponding EHR for the time period of observation regarding information on kidney injury. In addition, we evaluated the respective SmPCs of the OAT, which showed low frequencies of kidney injury/nephrotoxicity. Following that, we consider confounding by alternative reasons for eGFR_SCr_ decline in our study as limited.

## CONCLUSIONS

This study shows that in clinical routine a considerable number of oral antitumor therapeutics associated with pseudo‐worsening of kidney function are prescribed. However, very low usage of creatinine‐independent methods of kidney function assessment, such as eGFR based on cystatin C, indicates that further counseling of oncologists on oral antitumor therapeutic‐induced pseudo‐worsening of kidney function is required in order to improve patient safety. In addition, investigations on oral antitumor therapeutics, likely causing, but not yet proven to cause pseudo‐worsening of kidney function through prospective investigations are necessary to be able to differentiate between pseudo‐worsening of kidney function and real kidney injury in clinical routine care.

## FUNDING

The randomized, multicenter AMBORA trial was supported by the German Cancer Aid (project grant no. 70112447/70112457). The AMBORA Center was also supported by the German Cancer Aid (project grant no. 70114066/70114067). The present work was supported in part by the overarching use case of the German Medical Informatics Initiative ‘INTERPOLAR_MI – INTERventional POLypharmacy – drug interAction, Risks’ by the German Federal Ministry of Research, Technology and Space (BMFTR, 01ZZ2320B).

## CONFLICTS OF INTEREST

M.I.S. declares no conflict of interest. P.D. has received honoraria from AstraZeneca GmbH and reports other financial or nonfinancial interests (earmarked financial contribution: first award of the MSD Germany Health Award 2021). P.L. declares no conflict of interest. L.C. declares no conflict of interest. K.G. has received honoraria from AstraZeneca GmbH and Roche Pharma and reports other financial or nonfinancial interests (earmarked financial contribution: first award of the MSD Germany Health Award 2021). R.F. has received consulting fees and honoraria from Sennewald, AstraZeneca, MSD, Merck, Novocure, and Merck Serono. M.F.N. has received consulting fees from Janssen‐Cilag, BMA House, Pentax GmbH, S. Karger, Carpmael & Rensford, Boehringer Ingelheim International, Monte Rosa Therapeutics, TRex Bio, SciRhom, and Pfizer Biopharmaceuticals; honoraria from Janssen‐Cilag, Falk Foundation, Skaggs School of Pharmacy & Pharmaceutical Sciences, Medi K S.r.l., AOCC, Lilly, Northwell Foundation, and Takeda. B.W. has received speaking fees from Johnson & Johnson. M.P. has received consulting fees and honoraria from IPSEN, AAA, Novartis, ITM, Serb, Sanofi, Recordati, Esteve, Boehringer Ingelheim, MSD, Tairix, Medscape, MedUpdate; clinical trials with fees to the institution from Novartis, AAA, ITM, Boehringer Ingelheim, IPSEN. C.B. reports honoraria for consulting (advisory boards) and/or presentations at conferences from Almirall, BMS, MSD, Novartis, Immunocore, Pierre Fabre, Regeneron, and Sanofi, outside the submitted work. M.W.B. declares no conflict of interest. A.M. declares no conflict of interest. F.D. has received consulting fees from Lilly and Sandoz‐Hexal; honoraria from Johnson & Johnson and Gilead; and reports other financial or nonfinancial interests (earmarked financial contribution: first award of the MSD Germany Health Award 2021). M.F.F. has received grants or contracts from Boehringer Ingelheim and Heidelberg Pharma Research GmbH; consulting fees and honoraria from Boehringer Ingelheim; and reports other financial or nonfinancial interests (earmarked financial contribution: first award of the MSD Germany Health Award 2021).

## AUTHOR CONTRIBUTIONS

M.I.S., P.D., P.L., K.G., L.C., R.F., M.F.N., B.W., M.P., C.B., M.W.B., A.M., F.D., and M.F.F. wrote the manuscript. M.I.S., P.D., and M.F.F. designed the research. M.I.S. and P.D. performed the research. M.I.S. analyzed the data.

## Supporting information


Data S1:
