# Supplementary material for: Extent and Incidence of Pseudo‐Worsening of Kidney Function Due to Oral Antitumor Therapeutics in the AMBORA Cohort: An Analysis of Real‐World Data
Source: Clin Pharmacol Ther. 2025 Dec 22;119(3):802–11. doi: 10.1002/cpt.70166 (PMC12882744; doi:10.1002/cpt.70166)
Supplement: Supplementary file 1 — Data S1: [file CPT-119-802-s001.pdf]

Supplementary Material

Extent and incidence of pseudo-worsening of kidney function due to oral antitumor therapeutics in the AMBORA cohort: an analysis of real-world data

Michael I. Sponfeldner[1], Pauline Dürr[1,2,3,4], Phyllis Lensker[1,2,3,4], Katja Gessner[1,3,4], Lisa Cuba[1,2,3,4,12], Rainer Fietkau[3,4,5], Markus F. Neurath[3,4,6], Bernd Wullich[3,4,7], Marianne Pavel[3,4,6], Carola Berking[3,4,8], Matthias W. Beckmann[3,4,9], Andreas Mackensen[3,4,10], Frank Dörje[2,3,4,11], and Martin F. Fromm[1,3,4,11]

[1]Institute of Experimental and Clinical Pharmacology and Toxicology, Friedrich-Alexander-Universität Erlangen-Nürnberg, Erlangen, Germany

[2]Pharmacy Department, Erlangen University Hospital and Friedrich-Alexander-Universität Erlangen-Nürnberg, Erlangen, Germany

[3]Comprehensive Cancer Center Erlangen-EMN, Uniklinikum Erlangen, Erlangen, Germany

[4]Bavarian Cancer Research Center (BZKF), Erlangen, Germany

[5]Department of Radiation Oncology, Uniklinikum Erlangen and Friedrich-Alexander-Universität Erlangen-Nürnberg, Erlangen, Germany

[6]Department of Medicine 1, Gastroenterology, Pneumology and Endocrinology, Uniklinikum Erlangen and Friedrich-Alexander-Universität Erlangen-Nürnberg, Erlangen, Germany

[7]Department of Urology and Pediatric Urology, Uniklinikum Erlangen and Friedrich-Alexander-Universität Erlangen-Nürnberg, Erlangen, Germany

[8]Department of Dermatology, Uniklinikum Erlangen and Friedrich-Alexander-Universität Erlangen-Nürnberg, Erlangen, Germany

[9]Department of Obstetrics and Gynecology, Uniklinikum Erlangen and Friedrich-Alexander-Universität Erlangen-Nürnberg, Erlangen, Germany

[10]Department of Internal Medicine 5, Hematology and Oncology, Uniklinikum Erlangen and Friedrich-Alexander-Universität Erlangen-Nürnberg, Erlangen, Germany

[11]FAU NeW – Research Center New Bioactive Compounds, Friedrich-Alexander-Universität Erlangen-Nürnberg, Erlangen, Germany

[12]Pharmacy Department, Clinic Floridsdorf, Vienna Healthcare Group, Vienna, Austria (present address)

Table of contents

1. Table S1 Frequencies of all oral antitumor therapeutics used in the 238 patients treated with oral antitumor therapeutics likely causing or proven to cause pseudo-worsening of kidney function
2. Table S2 Baseline characteristics of the comparison cohort of patients treated with oral antitumor therapeutics unlikely to cause pseudo-worsening of kidney function (n=67)
3. Table S3 Comparison of serum creatinine concentrations / serum creatinine-based eGFR and cystatin C / cystatin C-based eGFR measurements in the 238 patients treated with oral antitumor therapeutics likely causing or proven to cause pseudo-worsening of kidney function
4. Figure S1 Flow diagram for the 67 included patients in the comparison cohort treated with oral antitumor therapeutics unlikely to cause pseudo-worsening of kidney function
5. Figure S2 Analysis of mean decrease in serum creatinine-based eGFR in 67 patients newly treated with oral antitumor therapeutics unlikely to cause pseudo-worsening of kidney function within 30 days of oral antitumor therapeutic initiation.
6. Figure S3 Serum creatinine-based eGFR in 238 patients newly treated with oral antitumor therapeutics likely causing or proven to cause pseudo-worsening of kidney function before oral antitumor therapeutic initiation and within 30 days, stratified for baseline eGFR levels. A. baseline eGFR > 90 ml/min; B. baseline eGFR 89-60 ml/min; C. baseline eGFR 59-30 ml/min.
7. Figure S4 Serum creatinine changes in patients newly treated with oral antitumor therapeutics likely causing or proven to cause pseudo-worsening of

kidney function stratified for individual oral antitumor therapeutics (≥3 patients / OAT in the population of 238 patients).

8. Figure S5 Serum creatinine-based eGFR changes in patients newly treated with oral antitumor therapeutics likely causing or proven to cause pseudo-worsening of kidney function stratified for individual oral antitumor therapeutics (< 3 patients / OAT in the population).
9. Figure S6 Changes in serum creatinine-based eGFR in 238 patients newly treated with oral antitumor therapeutics likely causing or proven to cause pseudo-worsening of kidney function before oral antitumor therapeutic initiation, within 30 days and from day 31 to week 12.

Table S1 Frequencies of all oral antitumor therapeutics used in the 238 patients treated with oral antitumor therapeutics likely causing or proven to cause pseudo-worsening of kidney function

OAT	Patients [n]
abemaciclib†	15
alectinib	1
apalutamide	2
avapritinib	1
axitinib	4
brigatinib	1
cabozantinib†	32
cobimetinib	1
crizotinib†	3
dabrafenib	28
darolutamide	7
dasatinib	1
encorafenib	9
erlotinib	1
ibrutinib	2
imatinib†	5
lenvatinib	8
lorlatinib	1
midostaurin	12
nilotinib	9
niraparib†	3
olaparib†	15
osimertinib	6
palbociclib†	14
pazopanib	10
pralsetinib	1
regorafenib	3
ribociclib	23
ruxolitinib	2
selpercatinib	1
sorafenib	2
sunitinib	6
tepotinib	1
tivozanib	3
trametinib	2
vandetanib†	1
vemurafenib†	1
zanubrutinib	1

† = OAT proven to cause pseudo-worsening of kidney function (Sponfeldner MI, et al. Pseudo-Worsening of Kidney Function Due to Inhibition of Renal Creatinine Secretion: Quality of Information Provided in Prescribing Information/SmPC. Clin Pharmacol Ther. 2024 Nov;116(5):1259-1268. doi: 10.1002/cpt.3374.; Zibetti Dal Molin G, Westin SN, Msaouel P, Gomes LM, Dickens A, Coleman RL. Discrepancy in calculated and measured glomerular filtration rates in patients treated with PARP inhibitors. Int J Gynecol Cancer. 2020 Jan;30(1):89-93. doi: 10.1136/ijgc-2019-000714.). Abbreviations: OAT = oral antitumor therapeutic

Table S2 Baseline characteristics of the comparison cohort of patients treated with oral antitumor therapeutics unlikely to cause pseudo-worsening of kidney function (n=67)

characteristic	no. [%]
age [years] (mean, range)	66.1 (21-88)
sex [female]	18 [26.9%]
**cancer type**	
**solid tumors**	
prostate cancer	21 [31.3%]
neuroendocrine tumors (small intestine, lung, pancreas)	11 [16.4%]
colorectal carcinoma	7 [10.4%]
small intestine carcinoma	3 [4.5%]
pancreatic carcinoma	3 [4.5%]
basal cell carcinoma	2 [3.0%]
gastric carcinoma	2 [3.0%]
cholangiocarcinoma	2 [3.0%]
renal cell carcinoma	2 [3.0%]
breast cancer	1 [1.5%]
glioblastoma	1 [1.5%]
soft tissue sarcoma	1 [1.5%]
**hematologic malignancies**	
multiple myeloma	5 [7.5%]
acute myeloid leukemia	4 [6.0%]
acute promyelocytic leukemia	1 [1.5%]
polycythaemia vera	1 [1.5%]
**oral antitumor therapeutic**	
abiraterone	16 [23.9%]
everolimus	16 [23.9%]
trifluridine	9 [23.9%]
capecitabine	5 [7.4%]
enzalutamide	4 [5.9%]
venetoclax	4 [5.9%]
lenalidomide	2 [2.9%]
pomalidomide	2 [2.9%]
hydroxycarbamide	1 [1.5%]
lomustine	1 [1.5%]
panobinostat	1[1.5%]
sonidegib	1 [1.5%]
tegafur, gimeracil & oteracil	1 [1.5%]
temozolomide	1 [1.5%]
tretinoin	1 [1.5%]
trofosfamide	1 [1.5%]
vismodegib	1 [1.5%]
**$eGFR_{SCr}$**	
baseline [ml/min] (mean, range)	87.7 (32.5-11.8)
baseline ≥90 ml/min	40 [59.7%]
baseline 60 - 89 ml/min	18 [26.9%]

baseline 30- 59 ml/min	9 [13.4%]

Table S3 Comparison of serum creatinine concentrations / serum creatinine-based eGFR and cystatin C / cystatin C-based eGFR measurements in the 238 patients treated with oral antitumor therapeutics likely causing or proven to cause pseudo-worsening of kidney function

Patient	OAT	Age	Measurement	SCr [mg/dl]	$eGFR_{SCr}$ [ml/min]	CysC [mg/dl]	$eGFR_{CysC}$ [ml/min]
1	abemaciclib	71	1*	1.30	41	1.05	65
			2*	1.46	36	1.18	56
2	abemaciclib	75	1*	2.04	23	1.84	30
3	abemaciclib	66	1*	1.29	43	0.79	96
4	crizotinib	77	1*	0.78	78	0.80	91
5	darolutamide	64	1*	1.34	56	1.17	62
6	niraparib	72	1*	0.98	58	1.05	65
7	osimertinib	75	1	0.78	74	1.56	38
8	osimertinib	72	1*	0.67	93	0.95	110
9	pazopanib	75	1	0.90	72	1.03	66
10	ribociclib	66	1	1.31	96	1.49	42

* = measurements with higher $eGFR_{CysC}$ than $eGFR_{SCr}$. Abbreviations = SCr = serum creatinine concentration, CysC = serum cystatin C concentration, $eGFR_{SCr}$ = eGFR calculated based on serum creatinine concentration, $eGFR_{CysC}$ = eGFR calculated based on serum cystatin C concentration, OAT = oral antitumor therapeutic

Figure S1 Flow diagram for the 67 included patients in the comparison cohort treated with oral antitumor therapeutics unlikely to cause pseudo-worsening of kidney function

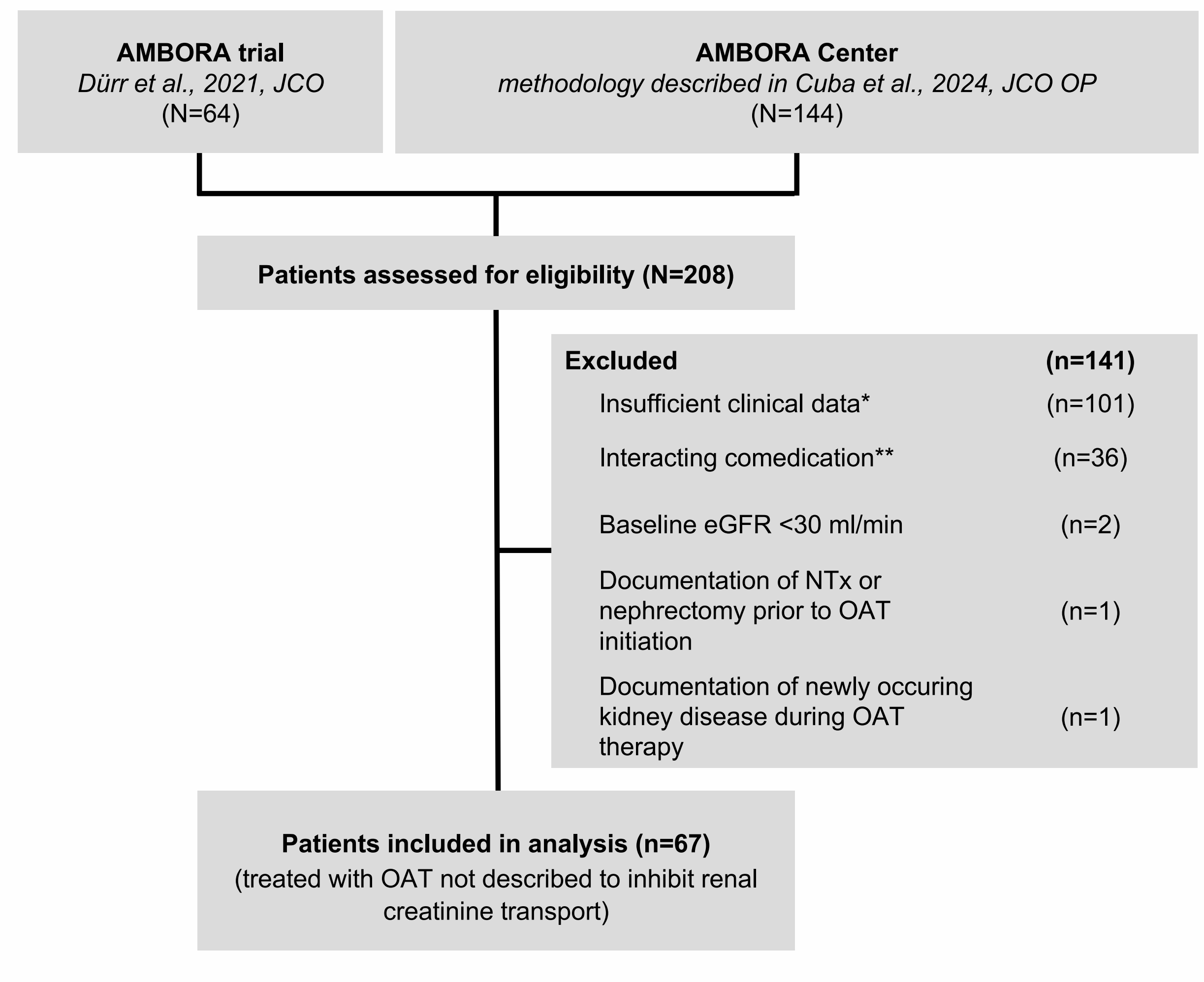

* = insufficient clinical data corresponding to no serum creatinine concentration available at baseline, no follow-up serum creatinine concentration available, < 2 serum creatinine concentrations available within 7 days of initiation of the oral antitumor therapeutic, no baseline co-medications available; ** = comedications also described to cause pseudo-worsening of kidney function (e.g., trimethoprim) or being nephrotoxic. Abbreviations: eGFR = estimated glomerular filtration rate, OAT = oral antitumor therapeutic, NTx = kidney transplantation, SCr = serum creatinine concentration.

Figure S2 Analysis of mean decrease in serum creatinine-based eGFR in 67 patients newly treated with oral antitumor therapeutics unlikely to cause pseudo-worsening of kidney function within 30 days of oral antitumor therapeutic initiation

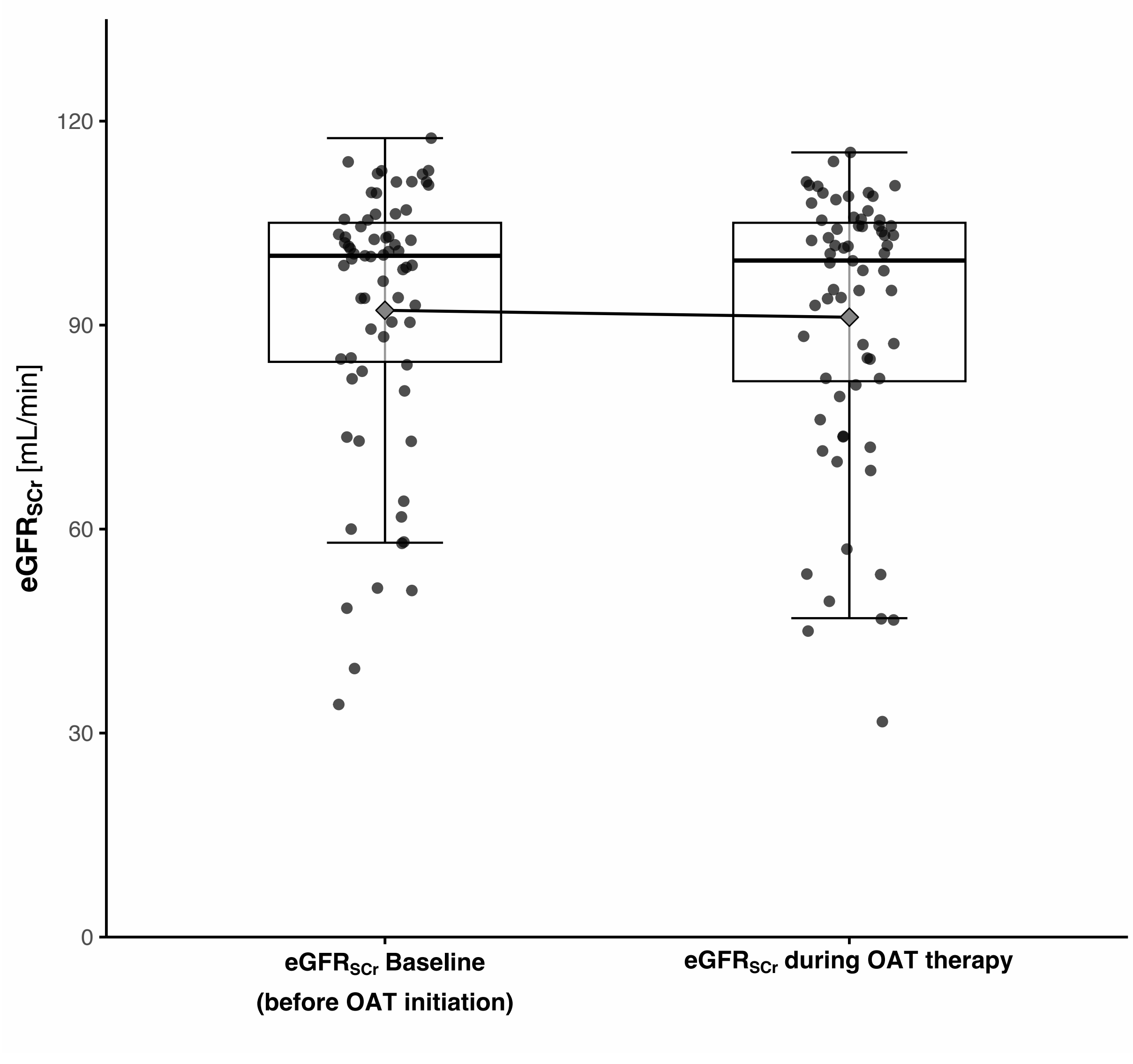

Box plot: dots, data for individual patients with random jittering for better graphical representation; grey square, mean. Abbreviations: eGFR = estimated glomerular filtration rate, OAT = oral antitumor therapeutic, SCr = serum creatinine concentration. *** P<0.001

Figure S3 Serum creatinine-based eGFR in 238 patients newly treated with oral antitumor therapeutics likely causing or proven to cause pseudo-worsening of kidney function before oral antitumor therapeutic initiation and within 30 days, stratified for baseline eGFR levels. A. baseline eGFR > 90 ml/min; B. baseline eGFR 89-60 ml/min; C. baseline eGFR 59-30 ml/min.

A

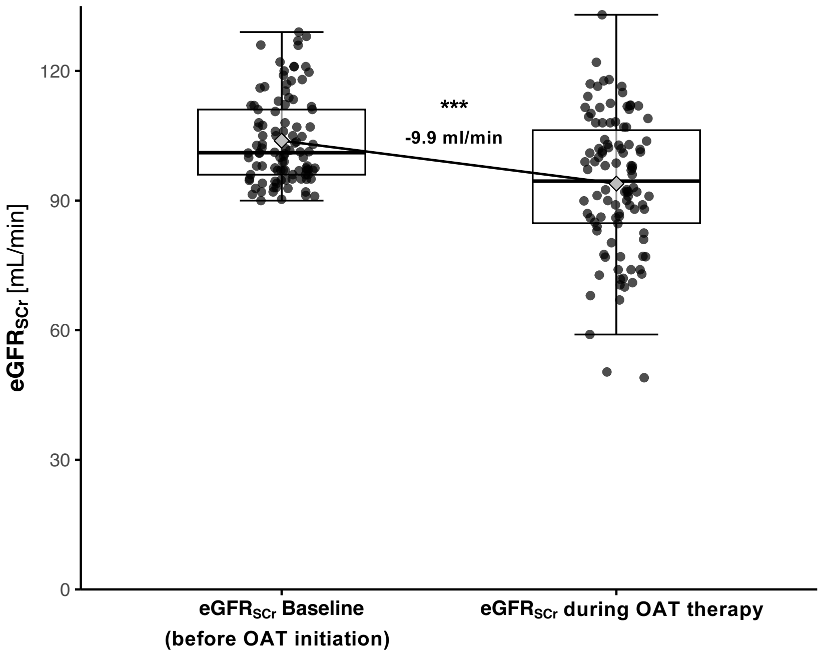

B

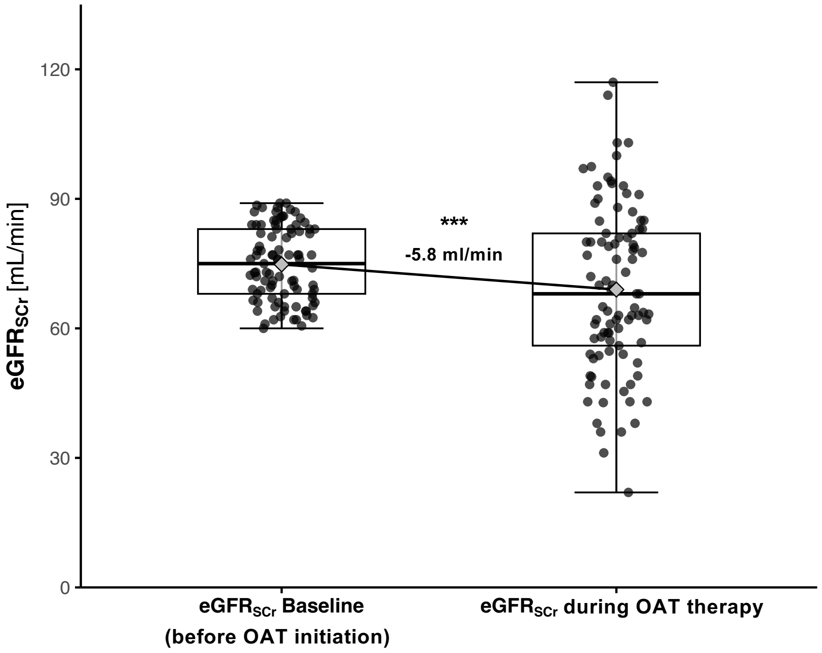

C

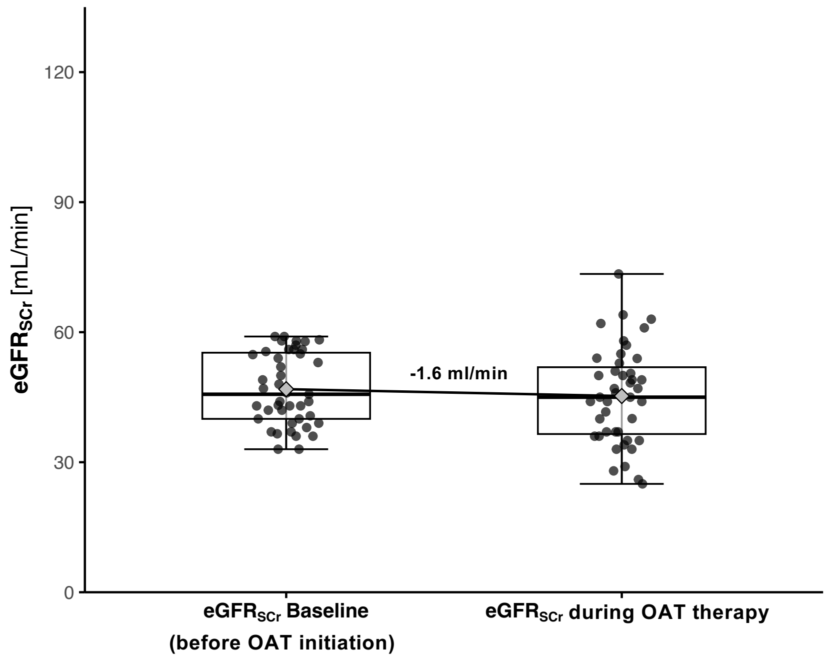

Box plot: dots, data for individual patients with random jittering for better graphical representation; grey square, mean. Abbreviations: eGFR = estimated glomerular filtration rate, OAT = oral antitumor therapeutic, SCr = serum creatinine concentration. *** $P<0.001$

Figure S4 Serum creatinine changes in patients newly treated with oral antitumor therapeutics likely causing or proven to cause pseudo-worsening of kidney function stratified for individual oral antitumor therapeutics (≥3 patients / OAT in the population of 238 patients)

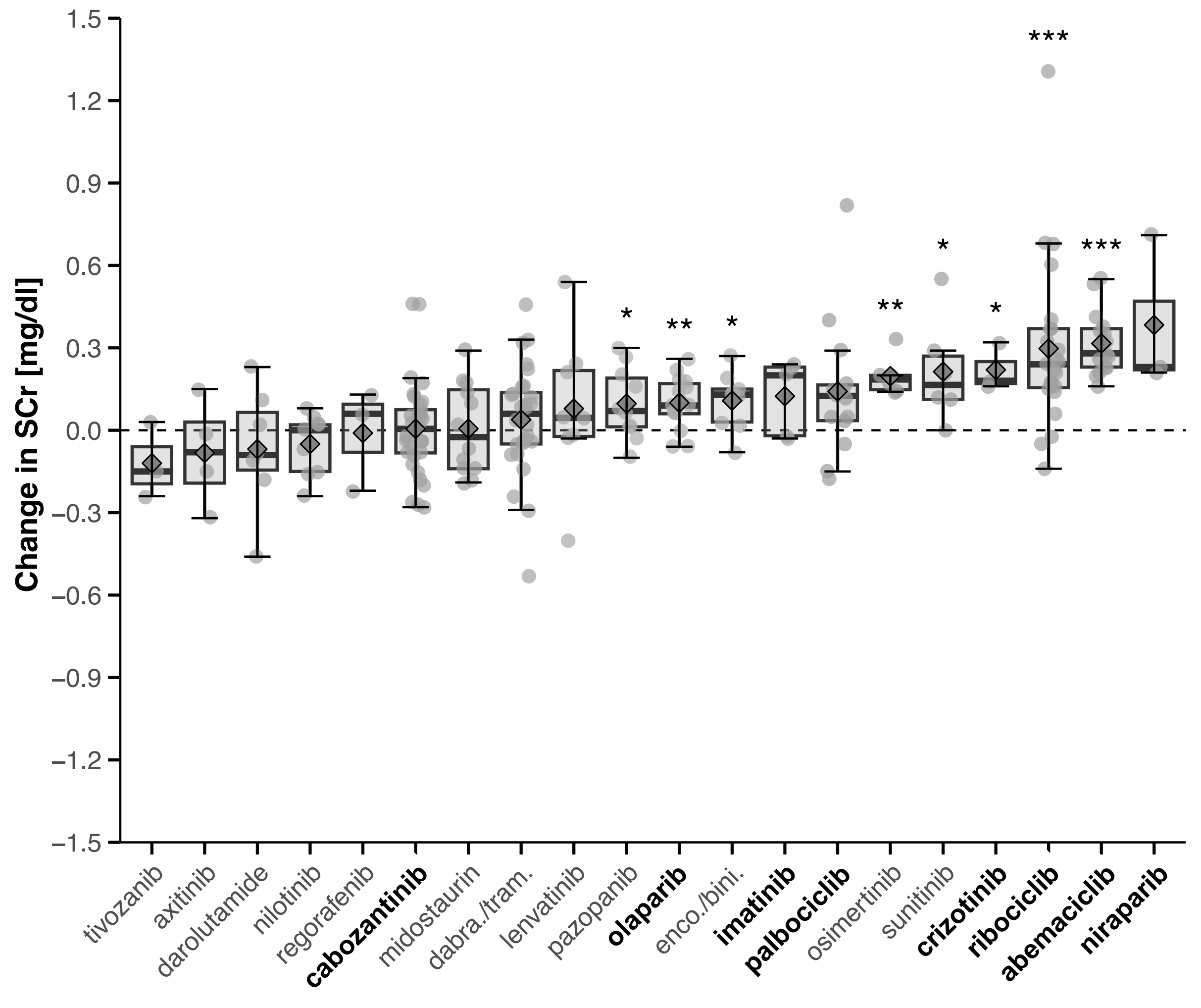

OAT in bold are proven to cause pseudo-worsening of kidney function. Box plot: dots, data for individual patients with random jittering for better graphical representation; grey square, mean. Abbreviations: eGFR = estimated glomerular filtration rate, OAT = oral antitumor therapeutic, SCr = serum creatinine concentration. * P<0.05, ** P<0.01, *** P<0.001

Figure S5 Serum creatinine-based eGFR changes in patients newly treated with oral antitumor therapeutics likely causing or proven to cause pseudo-worsening of kidney function stratified for individual oral antitumor therapeutics (< 3 patients / OAT in the population)

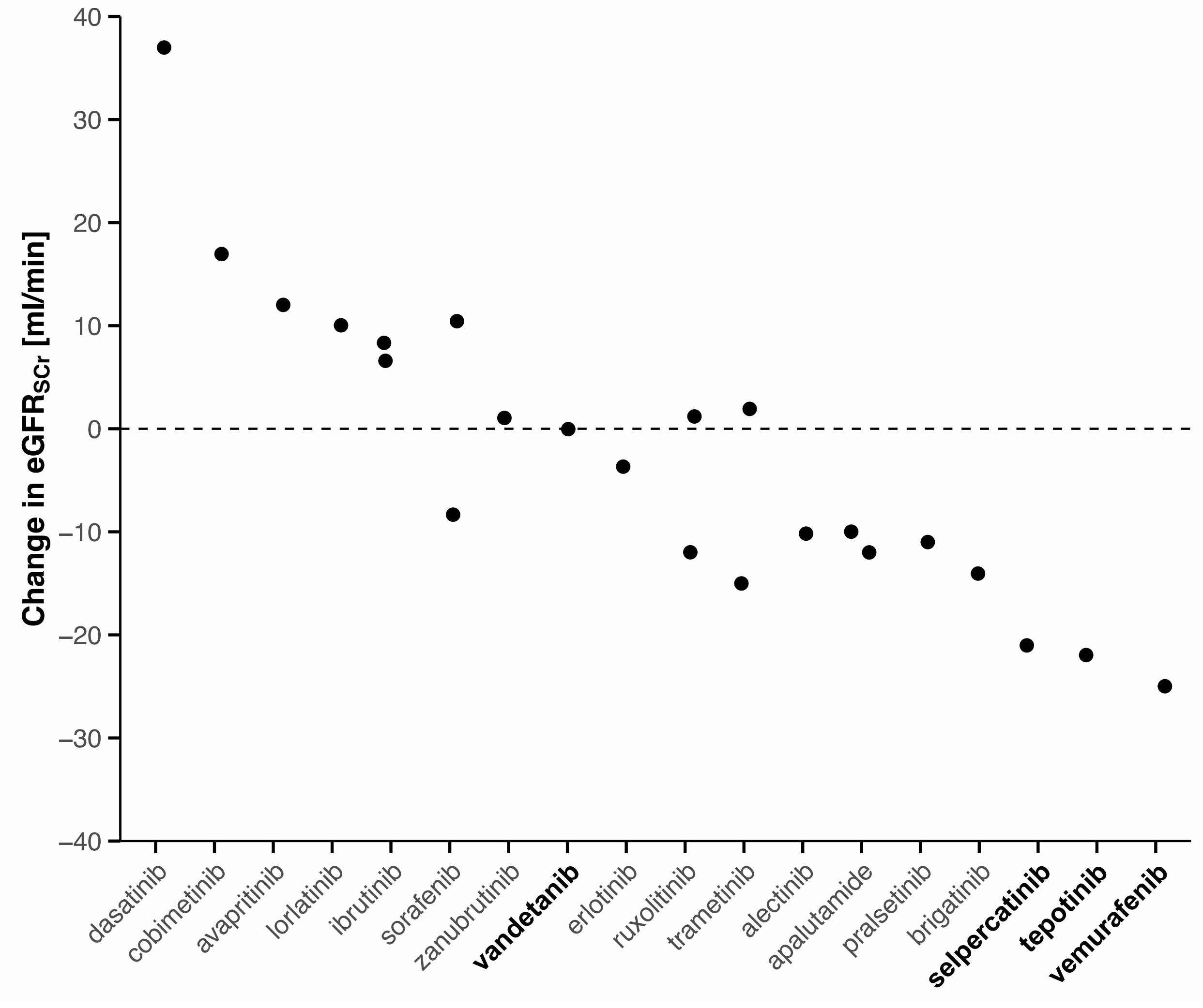

OAT in bold are proven to cause pseudo-worsening of kidney function. Dots, data for individual patients; Abbreviations: eGFR = estimated glomerular filtration rate, OAT = oral antitumor therapeutic, SCr = serum creatinine concentration.

Figure S6 Changes in serum creatinine-based eGFR in 238 patients newly treated with oral antitumor therapeutics likely causing or proven to cause pseudo-worsening of kidney function before oral antitumor therapeutic initiation, within 30 days and from day 31 to week 12.

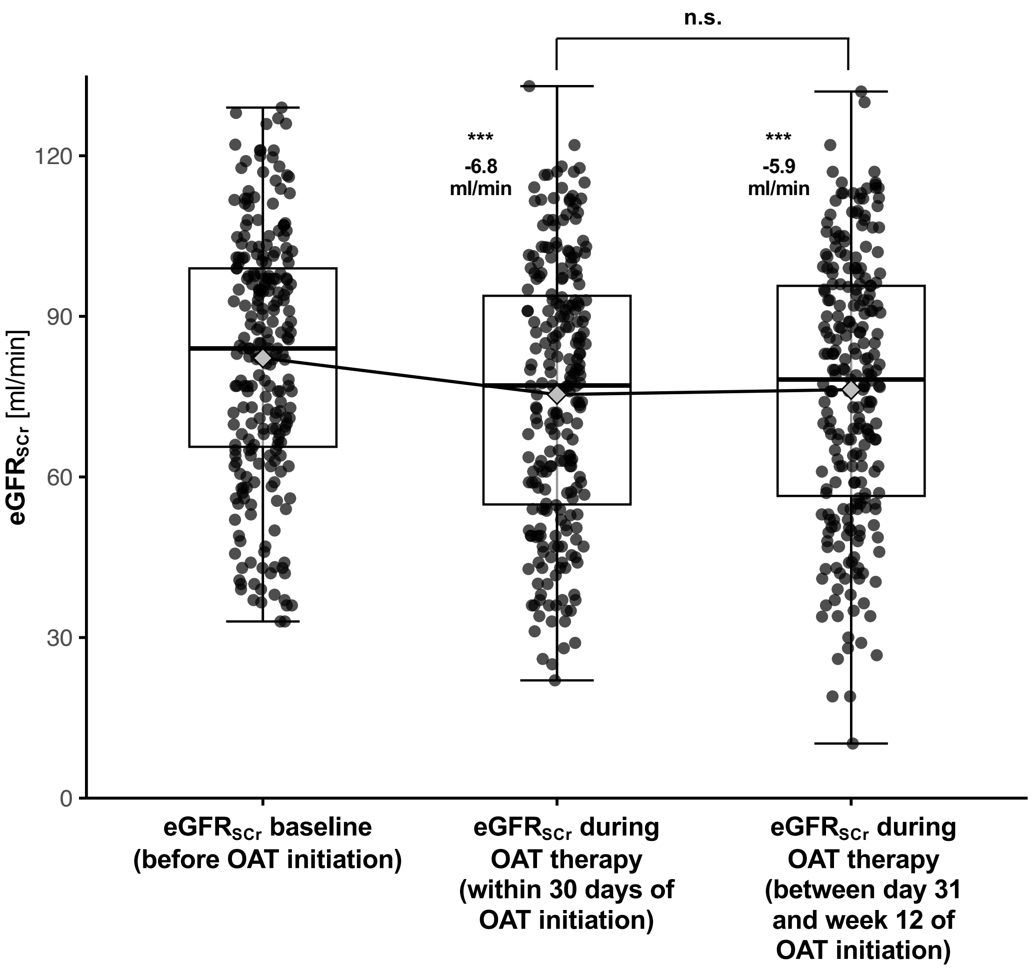

Box plot: dots, data for individual patients with random jittering for better graphical representation; grey square, mean. Abbreviations: eGFR = estimated glomerular filtration rate, OAT = oral antitumor therapeutic, SCr = serum creatinine concentration. *** P<0.001, statistical significance was calculated in comparison to baseline.
